# Ocular temperature measured by infrared thermography in dogs varies with biological and environmental factors

**DOI:** 10.3389/fvets.2026.1849602

**Published:** 2026-09-03

**Authors:** Sahar Rostami, Kimberly A. Woodruff, Abigail McBride, Jingyi Shi, David R. Smith

**Affiliations:** 1 Department of Pathobiology and Population Medicine, College of Veterinary Medicine, Mississippi State University, Starkville, MS, United States; 2 Department of Mathematics and Statistics, College of Arts and Sciences, Mississippi State University, Starkville, MS, United States

**Keywords:** canine, eye temperature, infrared thermography, mixed-effects models, rectal temperature, shelter dogs

## Abstract

Non-contact temperature assessment is highly valuable for screening animal populations, but its performance can be influenced by various external and biological variables. This study aimed to quantify the association between eye temperature (ET) and rectal temperature (RT) in dogs, and to identify physiological and environmental factors associated with these readings. Field study: ET was obtained with a thermal camera up to 3 times per visit (~10-min intervals), followed by RT measurement. Eye temperature was defined as the highest temperature recorded from either eye. A linear mixed-effects model evaluated ET in relation to capture time (1–3), RT, age, sex, body size, coat length, ambient temperature, and relative humidity. Device temporal stability assessment: To account for potential camera drift observed in our previous research, temperature at a fixed target inside an insulated box was recorded every 5 min for 1 h and analyzed with repeated-measures mixed model regression. Results. We analyzed 129 observations from 78 dogs. In the final ET model, both RT (*β* = 0.77 °C; 95% C.I. 0.512–1.019 °C) and humidity (*β* = 0.01 °C; 95% C.I. 0.002–0.013 °C) were positively and significantly associated with ET. The device stability assessment demonstrated no significant time-related measurement drift. Overall, ET was associated with RT but was not interchangeable and ET was slightly affected by environmental humidity. These results support further investigation of using ET as a rapid, noncontact temperature monitoring procedure.

## 1 Introduction

Veterinary medicine in group-housing environments operates at the interface of individual patient care and population health. High intake, frequent turnover, and close confinement increase contact rates and environmental pathogen loads, which increase the risk of infectious disease transmission and stress-related morbidity in dogs (1, 2). In that operational setting, it is desirable for staff to screen many animals for evidence of disease quickly, often during intake or daily rounds, and make decisions about animal health status with limited handling time. Temperature assessment is central to those health screening practices because hypothermia, fever (pyrogen-mediated set-point elevation) and hyperthermia (uncontrolled heat gain/limited dissipation) signal conditions that require immediate management or isolation. Whereas rectal temperature (RT) is widely treated as the practical reference for estimating canine core temperature, its application can be constrained by procedure time, animal resistance, welfare considerations, and biosecurity burden; non-rectal approaches may improve output but might vary in accuracy (3–5).

Infrared thermography (IRT) is a screening tool that senses thermal radiation emitted from surfaces and converts it to temperature, providing rapid, noncontact measurements over a user-defined region of interest (ROI) (6, 7). The ocular region is often targeted because its tissues are highly perfused, and less covered by hairs/furs, potentially reducing thermal lag relative to haired skin (8, 9). Ocular IRT could help identify dogs that require confirmatory evaluation. In practice, IRT is a surface measurement: observed temperatures reflect emissivity, reflected radiation, and ambient conditions (air temperature, humidity) (6, 7, 10).

The evidentiary base for eye temperature (ET) as a proxy for RT in dogs is promising but inconsistent. In a 300-dog clinical study using a noncontact infrared device at the cornea, ocular readings were consistently lower than rectal values and showed moderate correlation; the authors concluded the methods were not interchangeable but that ocular measures tracked directional changes in thermal status (9). In exercising dogs, eye and ear temperatures moved with RT across activity states, although ear temperature tended to align more closely with rectal values at rest and post-exercise, and ocular values showed greater dispersion in some conditions (11). Device temporal stability assessment was initiated because in our previous work, conducted with a different camera, we observed a steady reduction in temperature values during sequential infrared captures (12). This finding suggested the possibility of internal measurement drift within the specific camera model. The main objective of this study was to evaluate the practical use of ocular thermography in a real-world veterinary environment. Because the eye and the rectum naturally operate at different temperature ranges, we did not attempt to calculate a perfect one-to-one agreement between the two methods. Rather, we wanted to see if shifts in ET reliably track alongside shifts in RT when using a fast, low-stress imaging method that is easy to perform. Additionally, we sought to identify how animal physical characteristics and camera behavior affect these repeated readings. Ultimately, this approach provides a realistic understanding of how thermal imaging can be utilized as an initial, restraint-free screening tool.

## 2 Materials and methods

### 2.1 Field study: ocular thermography in shelter dogs

Animal procedures were conducted under approval of the Mississippi State University IACUC, and shelter management provided consent for participation of shelter-owned dogs. We conducted an observational study in an animal shelter in Mississippi, with data collection during routine operating hours in the afternoon (12 p.m. to 4 p.m.). Data were collected while dogs remained in their kennels to minimize handling and preserve real-world screening conditions. Dogs were eligible based on brief observation: normal alertness and posture, no lethargy, no persistent coughing or sneezing, and no visible nasal or ocular discharge. Dogs could be measured during multiple shelter visits; each dog had a unique identifier to enable linkage across sessions. A visit was defined as a single measurement session for an individual dog (up to 3 ET captures obtained approximately 10 min apart during 1 afternoon round).

At the start of each imaging session, ambient temperature and relative humidity were recorded using a combined digital thermometer–hygrometer (INKPET; stated accuracy ±1.0 °C and ±5% RH). These values were entered into the data set and also applied to the thermal camera’s environmental settings.

Eye surface temperatures were obtained using a Thermal camera (Fotric 347A; IR resolution 384 × 288 pixels; stated noise-equivalent temperature difference ≈0.03 °C at 30 °C; measurement range −20 °C to 1,550 °C; accuracy ±2 °C or 2% at 25 °C ambient). Before imaging, the camera was allowed to warm up according to the manufacturer’s manual. The emissivity parameter was set to 0.98, and the working distance was standardized to ~60 cm. Dogs remained inside their kennels, and images were obtained from the aisle outside the kennel. For each enrolled dog, three ET images were collected approximately 10 min apart within a visit to assess repeatability. The primary anatomical target was the eyes.

Following the final ET reading of each visit, RT was measured without removing the dog from its kennel. To ensure safety and minimize stress, we gently held the dog in a standing position. A standard digital veterinary thermometer was prepared with a medical lubricant and carefully inserted into the rectum to a consistent depth. The device was held steadily in place until it produced an audible beep, indicating that a stable and final temperature reading had been successfully captured. This process was standardized for every animal. The following covariates were recorded: age (pup/adult), sex, size (small/medium/large), and coat length (short/medium/long).

**Figure 1:**
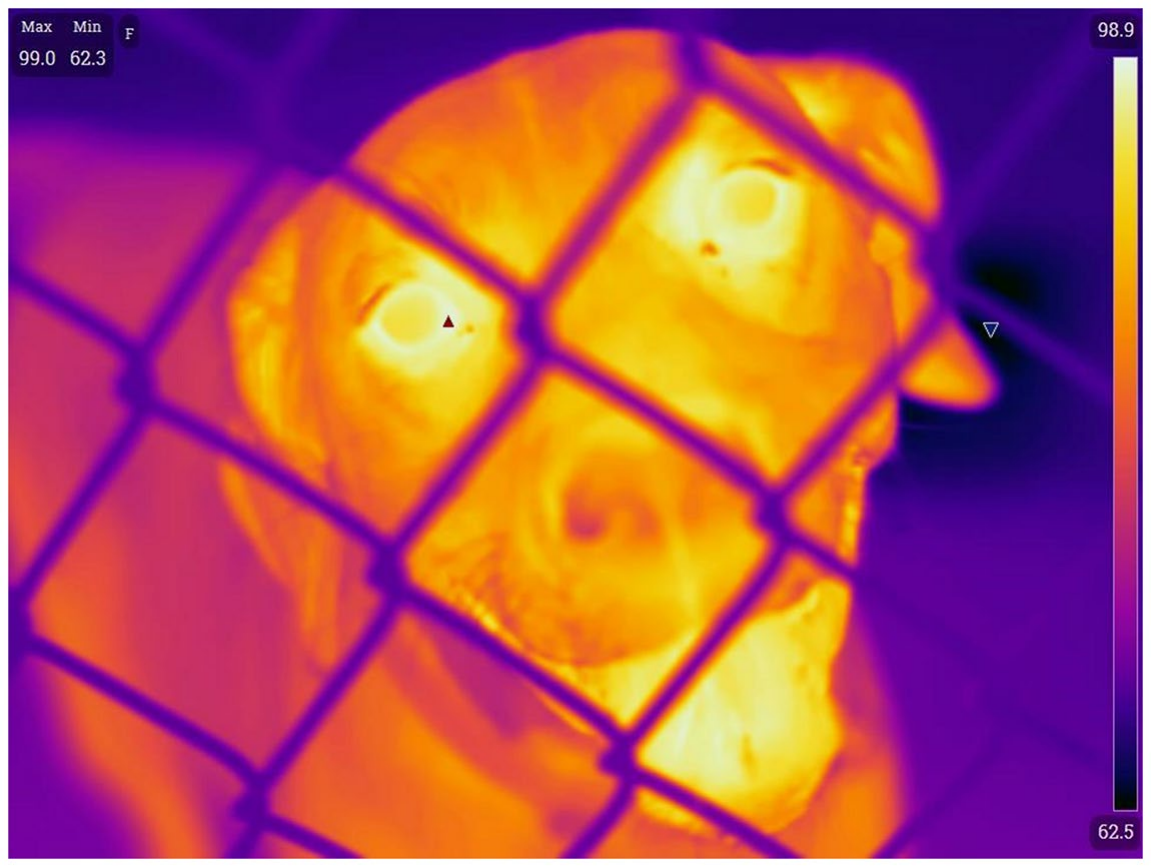
Representative eye temperature (ET) of a shelter dog. Thermal image of an adult male shelter dog taken from the kennel aisle at a standardized distance (~60 cm). Rather than manually outlining a geometric region of interest (ROI), the maximum temperature value from a single highest pixel across the visible ocular area of either eye was recorded as the ET to ensure rapid, objective data extraction. In this representative image, the highest temperature is indicated by the red triangle located over the dog’s right eye.

Images were processed using AnalyzIR Mercury software. Strict image inclusion and exclusion rules were applied prior to data extraction. Images were excluded if they were blurred, heavily obstructed by kennel bars, or exhibited overt surface reflections or sensor artifacts. To be included in the dataset, at least one eye had to be clearly visible and unobscured. By prioritizing these high-quality images, extracting the maximum temperature value provided a rapid, standardized, and objective metric that eliminated operator bias and the procedural delay required to manually draw a geometric ROI on moving dogs in kennel environments. For each image, the maximum ET recorded in either eye was extracted for analysis (Figure 1). For repeated measurements within a visit, observations were indexed by time (1, 2, and 3 corresponding to the three approximately 10-min captures).

### 2.2 Device temporal stability assessment

We conducted this part to assess the temporal stability of the thermal device, we created a shielded test environment using two insulated boxes (each roughly 39 × 32 × 30 cm) that were taped together. The internal surfaces were lined with paper, and the camera’s emissivity was adjusted to 0.93 as recommended by the operating manual. We placed this system in a draft-free indoor room with a steady temperature. By using an insulated system, we could protect the target from external fluctuations to specifically evaluate the camera’s internal performance. Once the device completed its warm-up phase, we monitored a single spot on the inner wall, taking readings every 5 min for 1 h. This 60-min trial was performed six times, with a 1-h interval between repetitions to ensure the camera hardware could properly cool down. The primary objective was to observe if the device produced a consistent measurement drift, rather than assessing its absolute temperature accuracy.

### 2.3 Statistical analysis

Linear mixed-effects regression models with repeated measures were used to analyze the data using MIXED procedure in SAS version 9.4 (SAS Institute Inc., Cary, NC, USA). Model development proceeded in stages, depending on the specific temperature outcome of interest. Before mixed-model fitting, candidate fixed effects were screened for collinearity by (i) pairwise Spearman correlation and (ii) design-matrix diagnostics from PROC REG (variance inflation factor [VIF], tolerance, and the COLLIN table). We flagged pairs with ∣𝜌∣ > 0.50 and *p* < 0.05 for review. Following common practice, we considered tolerance < 0.10 (VIF > 10) as evidence of collinearity, and we examined the condition index and proportion of variance patterns: rows with condition index ≥ 30 that also show ≥ 0.5 variance proportions for ≥ 2 predictors indicate a problematic association among predictors (13, 14).

#### 2.3.1 Models with ET as the outcome

Two sets of mixed models were developed for ET as the dependent variable. The first model included RT as a key explanatory variable to evaluate the association between ET and RT, while accounting for potential confounders. The primary objective of this model was to quantify how changes in core body temperature (RT) were reflected in peripheral thermal responses (ET). Candidate fixed effects considered during model development included age (puppy vs. adult), sex, body size (small, medium, large), coat length (short, medium, long), ambient temperature, humidity, and time (three repeated infrared captures per visit).

A second ET model was constructed excluding RT as an independent variable. The purpose of this model was to characterize how environmental and individual animal factors independently influenced ET, without the dominant effect of RT that could mask smaller associations.

For both models, the repeated-measures structure accounted for sequential temperature captures (capture order 1, 2, and 3) nested within measurement sessions (visit ID) for each individual animal (dog ID). To account for temporal correlation between consecutive temperature readings taken within the same visit, we applied an autoregressive covariance structure of order 1 [AR(1)]. A random intercept was also included for the measurement sessions nested within dogs, allowing baseline ET to vary and accounting for potential clustering due to repeated measurements across visits.

#### 2.3.2 Model with RT as the outcome

A complementary model was developed using RT as the dependent variable, primarily for comparison with the ET models and to evaluate whether RT was independently and similarly influenced by environmental and animal-level covariates. Because only one RT was recorded per visit, random intercept for dog (ID) was used, which appropriately captured repeated sampling across visits. For this model, ET was not included as a predictor because the primary relationship between ET and RT had already been characterized in the first ET model. Including ET again as an independent variable in the RT model would be statistically redundant for our specific analytical objectives. Candidate fixed effects for the RT model included age (puppy vs. adult), sex, body size (small, medium, large), coat length (short, medium, long), ambient temperature, and humidity.

For all three models, a manual forward selection strategy was employed. To guard against data-driven overfitting, model selection at each stage was primarily guided by the Akaike Information Criterion (AIC), which penalizes for added complexity. Variables were retained in the final parsimonious model only if their inclusion improved overall model fit (lowered the AIC). Statistical significance for evaluating the retained fixed effects was set at *p* < 0.05. Model assumptions were evaluated by visual inspection of residual diagnostics in SAS, including residuals-versus-fitted plots for linearity and homoscedasticity, histograms of residuals, and *Q*–*Q* plots for normality.

#### 2.3.3 Device temporal stability assessment

To test for change of temperature over time, linear mixed-effects model was fitted with temperature as the outcome, and time as a repeated factor. Statistical significance was determined at *p* < 0.05.

## 3 Results

### 3.1 Field study

**Figure 2:**
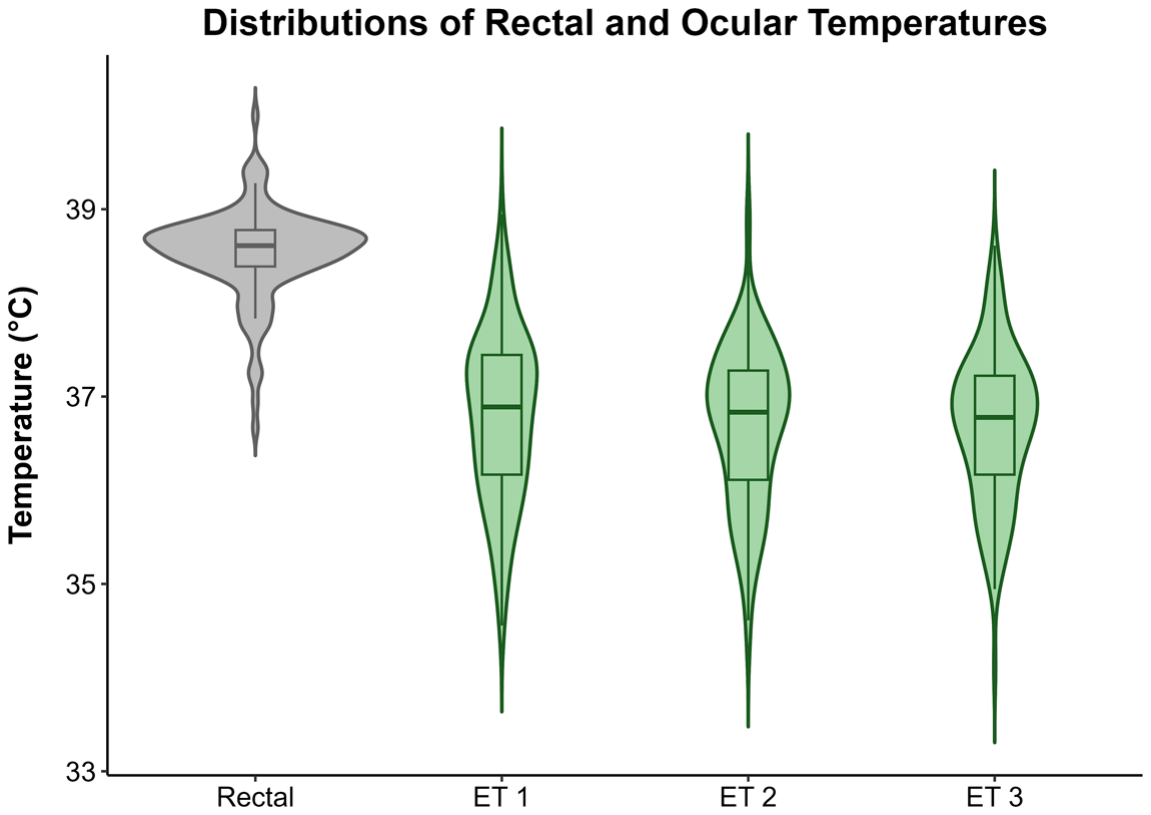
Exploratory visualization of raw temperature distributions. This figure presents an exploratory comparison of the raw, unadjusted distributions for rectal temperature (RT) and the three consecutive eye temperature (ET) measurements. Violin plots illustrate the density of the data, while the embedded boxplots indicate the median and interquartile range for each of the four distributions. Note that the *n* = 129 observations for each distribution represent repeated measurement sessions within the same cohort of dogs, rather than independent cross-sectional samples.

A total of 387 ET captures were obtained from 129 visits representing 78 unique shelter dogs, with 3 ET captures per visit (Table 1). Because the shelter environment is highly dynamic, animals frequently arrive and leave. We conducted our measurement sessions across multiple separate days, which allowed us to observe some dogs more than once if they were still housed at the facility. Consequently, this repeat sampling resulted in a higher total count of measurement visits compared to the count of individual dogs. Mean ET was recorded for the three within-visit repeats (Repeat 1: 36.8 °C; Repeat 2: 36.7 °C; Repeat 3: 36.7 °C), with overlapping 95% confidence limits (Table 2 and Figure 2). Within our study group, the measured RT spanned from a minimum of 36.7 °C up to a maximum of 40.0 °C. In mixed-effects models, capture order (1–3) was not associated with ET.

**Table 1:** Demographic characteristics of enrolled shelter dogs.

Characteristic	Category	Visits^a^ (*n* = 129)	Dogs^b^ (*n* = 78)
Age	Puppy	38	26
	Adult	91	52
Sex	Male	55	35
	Female	74	43
Size	Small	44	29
	Medium	62	39
	Large	23	10
Coat length	Short	89	59
	Medium	31	14
	Long	9	5

Values are counts.

a Represent measurement sessions (each session included up to 3 eye temperature captures).

b Represent unique animals.

**Table 2:** Mean eye temperature by capture order within visit.

Repeat	*N*	Mean ET (°C)	95% CI
1	129	36.8	36.6 to 37.0
2	129	36.7	36.5 to 36.8
3	129	36.7	36.5 to 36.8

Values summarize maximum eye temperature (ET) per capture, averaged across visits.

CI = Confidence interval.

ET = eye temperature.

All VIFs were < 2.0 and all tolerance > 0.50, indicating low collinearity by standard criteria. In the COLLIN table, no row with condition index ≥ 30 showed ≥ 0.5 variance proportions for two or more predictors. Pairwise Spearman correlations were generally weak to moderate; the only strong pair was age vs. size (𝜌 = 0.64, *p* < 0.001), reflecting expected biological overlap.

#### 3.1.1 Models with ET as the outcome

The first ET model retained RT and humidity as significant fixed effects. ET increased with RT (*β* = 0.77 °C, *p* < 0.001) and with humidity (*β* = 0.01 °C, *p* = 0.01; Table 3). In the ET model that excluded RT, relative humidity and body size were retained as fixed effects. Humidity remained a significant positive predictor (*β* = 0.01 °C, *p* = 0.01). This suggests that humidity has a consistent influence on peripheral surface temperature regardless of RT. Compared with small dogs (reference), mean ET was higher in medium dogs (*β* = 0.42 °C; *p* = 0.005) and large dogs (*β* = 0.63 °C; *p* = 0.001; Table 4).

**Table 3:** Fixed-effect estimates from mixed model of eye temperature including rectal temperature.

Effect	Estimate	*p* value	95% CI
Intercept	6.77	0.18	−3.101 to 16.649
RT (°C)	0.77	<0.001	0.512–1.019
Humidity (%)	0.01	0.013	0.002–0.013

RT = Rectal temperature.

CI = Confidence interval.

**Table 4:** Fixed-effect estimates from mixed model of eye temperature excluding rectal temperature.

Effect	Estimate	*p* value	95% CI
Intercept	35.92	<0.001	35.437–36.401
Large vs. small	0.63	0.001	0.247–1.021
Medium vs. small	0.42	0.005	0.125–0.711
Humidity (%)	0.01	0.014	0.002–0.014

Small body size was the reference category.

CI = confidence interval.

#### 3.1.2 Model with RT as the outcome

For comparison, a complementary mixed model was fit with RT as the dependent variable. The final RT model retained age and ambient temperature as significant fixed effects. Regarding age, RT was higher in adults compared with puppies (*β* = 0.4 °C, *p* < 0.001) and increased slightly with ambient temperature (*β* = 0.1 °C, *p* < 0.001; Table 5).

**Table 5:** Fixed-effect estimates from mixed model of rectal temperature.

Effect	Estimate	*p* value	95% CI
Intercept	36.41	<0.001	35.41–37.41
Age(adult)	0.4	<0.001	0.24–0.61
Age (puppy)	Reference	–	–
Ambient temperature (°C)	0.1	<0.001	0.04–0.12

CI = confidence interval.

Visual inspection of residual diagnostics (residuals vs. fitted plots, histograms, and *Q*–*Q* plots) did not reveal major departures from normality or constant variance.

### 3.2 Device temporal stability assessment

Across six replicated sessions (60 min each; measurements every 5 min), linear mixed-effects models found no evidence of temperature drift over time. The fixed effect for time (repeated factor across each session) was not statistically significant (*p* = 0.73). These findings support the temporal stability of the camera’s measurements over a 1-h operating period under controlled ambient conditions (a draft-free room with stable temperature and humidity, utilizing an insulated enclosure).

## 4 Discussion

By quantifying the long-wave infrared radiation, IRT provides a noncontact method for visualizing spatial thermal patterns (15). The speed and noninvasive nature of this imaging technique are key attributes that have encouraged its use in veterinary medicine. Its applications now span multiple species and include critical functions like disease surveillance, and initial screening or health assessment (16–23). The value of this technology lies in its ability to quickly identify animals with abnormal surface heat profiles from a distance, which can then prompt a physical examination.

The device stability assessment did not detect time-related drift in camera readings over 60 min. Eye temperature increased with rectal temperature and was also positively associated with humidity in models with and without rectal temperature. These results indicate that ET was sensitive to physiologic variation in body temperature under real-world conditions, but that environmental factors must be considered when interpreting ET results.

We chose to extract the single maximum pixel temperature from the visible ocular area rather than drawing a specific geometric ROI. Extracting the highest pixel temperature is a rapid, objective method well-suited for moving animals in kennels. However, a potential limitation of single-pixel extraction is that an isolated pixel may occasionally be susceptible to transient sensor noise or localized reflections, though our strict image exclusion criteria minimized this risk. While we recognize the practical advantages of this automated extraction, a limitation of this study is that we did not perform a direct, head-to-head validation comparing the diagnostic performance and repeatability of the single maximum pixel against a mean-temperature geometric ROI. Future studies should formally compare whether ROI-based measurements offer superior diagnostic accuracy or repeatability compared to maximum-pixel extraction in moving animals.

Capture order (captures 1–3) was tested during model development but was not retained in final models (*p* > 0.05, no AIC reduction), confirming high short-term repeatability across sequential ocular readings within a visit. Our findings are consistent with prior canine studies reporting positive associations between ET and RT and lower ocular temperatures than RT on average (11). The positive association between ET and RT aligns with experimental work in dogs demonstrating that non-contact thermography of the eye detects hyperthermia and correlates with RT both at rest and following exercise (24). It has been reported that ET underestimated RT at baseline but tracked exercise-induced increases with moderate to strong correlations, while tympanic measurements were closer to RT on average (11).

We also reported positive association between ET and humidity, independent of RT. Studies demonstrate that environmental moisture and tear-film dynamics meaningfully influence ET and its cooling rate between blinks (25–27). In dogs, field observations from racing greyhounds similarly reported higher ET with increasing humidity, reinforcing that moisture is a practical modifier in canine IRT (24).

In puppies, RT was modestly lower compared with adult dogs. This may reflect developmental differences in thermoregulatory capacity, metabolic rate, or body composition in younger animals. Ambient temperature was positively associated with RT, suggesting that even core body temperature may be influenced by environmental conditions. This highlights the importance of considering the environmental context when interpreting physiological measurements in dogs.

Previous research highlights that RT in dogs is influenced by both intrinsic and environmental factors. Some studies reported that smaller dogs exhibit slightly higher RT and higher ambient temperatures are associated with increased body temperature (28–30). These findings support the interpretation that biological and environmental factors should be considered when evaluating body temperature in dogs. Interestingly, our findings for ET diverged from patterns reported for core body temperature in previous studies. In our cohort, smaller dogs exhibited lower ET compared with larger dogs, whereas prior research consistently shows an inverse relationship between body size and core temperature, with smaller dogs having higher rectal temperatures than larger breeds (28, 29). This discrepancy may reflect differences in measurement type and physiology. Eye thermography, as a peripheral surface measurement, may be more sensitive to environmental heat loss and local blood flow, whereas RT reflects core thermal status. Additionally, vascularization of the ocular region could differentially modulate heat transfer in smaller versus larger dogs, potentially explaining the observed patterns. These results highlight that findings from peripheral temperature measurements may not directly match trends observed for core body temperature, and that surface measures can respond differently to body size and environmental conditions.

Our field conditions closely reflect real-world constraints, specifically by no handling and capturing images through the kennel from a consistent distance. In such contexts, ET offers several advantages: rapid non-contact application, compatibility with dogs that are difficult to handle, and the potential to flag animals for confirmatory assessment. Handling, stress, and physical activity may influence ocular surface temperature independent of fever (20, 24, 31). These factors can inflate false-positive rates if unaccounted for, especially in busy kennel aisles where their activity level may fluctuate with noise and human traffic. In our study, the measured RT ranged from 36.7 °C to 40.0 °C, a span that includes values both below and above the standard reference intervals commonly reported for dogs (32, 33). However, a primary limitation of this cohort is that it consisted of mainly healthy dogs, lacking clinically extreme febrile or hypothermic presentations. Therefore, the applicability of ET for the definitive detection of severe, clinically relevant temperature abnormalities requires further investigation. By capturing data that falls outside the typical normal range, we were able to evaluate the relationship between ocular and rectal temperatures across a broader physiological spectrum.

We set emissivity to 0.98 which aligns with veterinary thermography guidance for biological tissues, noting high emissivity of the ocular surface (34–36). We acquired images from the aisle to avoid handling. While this improves feasibility, it introduces potential challenges such as angle variation and intermittent blockage by kennel bars. We measured ambient temperature and humidity at each session. Prior work shows that wind, solar loading, and camera-to-object distance can materially affect ET, underscoring the value of site-level standardization and the process of capturing image (10, 37).

Taking together, both animal-level and environmental factors contribute to variation in RT and ET. While the magnitude of these effects is different, they should be considered when assessing health status in heterogeneous populations. Implementation studies should evaluate ET for abnormal temperature screening and validate performance across populations.

## Data Availability

The raw data supporting the conclusions of this article will be made available by the authors, without undue reservation.
